# Impact of phenolic-rich olive leaf extract on blood pressure, plasma lipids and inflammatory markers: a randomised controlled trial

**DOI:** 10.1007/s00394-016-1188-y

**Published:** 2016-03-07

**Authors:** Stacey Lockyer, Ian Rowland, Jeremy Paul Edward Spencer, Parveen Yaqoob, Welma Stonehouse

**Affiliations:** 1 Hugh Sinclair Unit of Human Nutrition, Department of Food and Nutritional Sciences, University of Reading, Reading, Berkshire RG6 6AP UK; 2 School of Food and Nutrition, College of Health, Massey University, Auckland, New Zealand; 3 Food and Nutrition Flagship, Commonwealth Scientific Industrial Research Organisation (CSIRO), Adelaide, Australia

**Keywords:** Olive leaf, Polyphenols, Cardiovascular disease, Blood pressure, Plasma lipids, Oleuropein

## Abstract

**Purpose:**

Dietary polyphenols have been demonstrated to favourably modify a number of cardiovascular risk markers such as blood pressure (BP), endothelial function and plasma lipids. We conducted a randomised, double-blind, controlled, crossover trial to investigate the effects of a phenolic-rich olive leaf extract (OLE) on BP and a number of associated vascular and metabolic measures.

**Methods:**

A total of 60 pre-hypertensive [systolic blood pressure (SBP): 121–140 mmHg; diastolic blood pressure (DBP): 81–90 mmHg] males [mean age 45 (±SD 12.7 years, BMI 26.7 (±3.21) kg/m^2^] consumed either OLE (136 mg oleuropein; 6 mg hydroxytyrosol) or a polyphenol-free control daily for 6 weeks before switching to the alternate arm after a 4-week washout.

**Results:**

Daytime [−3.95 (±SD 11.48) mmHg, *p* = 0.027] and 24-h SBP [−3.33 (±SD 10.81) mmHg, *p* = 0.045] and daytime and 24-h DBP [−3.00 (±SD 8.54) mmHg, *p* = 0.025; −2.42 (±SD 7.61) mmHg, *p* = 0.039] were all significantly lower following OLE intake, relative to the control. Reductions in plasma total cholesterol [−0.32 (±SD 0.70) mmol/L, *p* = 0.002], LDL cholesterol [−0.19 (±SD 0.56) mmol/L, *p* = 0.017] and triglycerides [−0.18 (±SD 0.48), *p* = 0.008] were also induced by OLE compared to control, whilst a reduction in interleukin-8 [−0.63 (±SD 1.13) pg/ml; *p* = 0.026] was also detected. Other markers of inflammation, vascular function and glucose metabolism were not affected.

**Conclusion:**

Our data support previous research, suggesting that OLE intake engenders hypotensive and lipid-lowering effects in vivo.

**Electronic supplementary material:**

The online version of this article (doi:10.1007/s00394-016-1188-y) contains supplementary material, which is available to authorized users.

## Introduction

Consumption of the so-called Mediterranean diet has been associated with a decreased risk of chronic diseases, in particular cardiovascular disease (CVD), when compared to other dietary regimes [1, 2]. These effects may be attributed, in part, to the olive oil (OO) component of the diet [3]. Research comparing refined OO to extra virgin OO (EVOO) has highlighted the biological activity of the (poly)phenol components contained within the water-soluble fraction of EVOO [4, 5]. In addition to the fruit (from which OO is derived), the leaves of the olive plant (*Olea europaea*) also contain phenolic compounds at a much higher concentration than those of the olive fruit and oil (1450 mg total phenolics/100 g fresh leaf [6] vs. 110 mg/100 g fruit [7] and 23 mg/100 ml EVOO [8]). The most abundant phenolic compounds present in the leaves are verbascoside, apigenin-7-glucoside, luteolin-7-glucoside, hydroxytyrosol (HT), tyrosol and the secoiridoid oleuropein, with secoiridoids being uniquely present in plants of the *Oleaceae* family [9].

Data emanating from a number of studies suggest that olive leaf extract (OLE) may influence CVD risk via its potential to induce anti-atherosclerotic, hypotensive, antioxidant, anti-inflammatory and hypocholesterolaemic effects (for review see [10]). The majority of these have been animal studies with limited data relating to effects in humans; however, human-derived data have begun to appear in the literature. OLE has been reported to lower systolic blood pressure (SBP) and diastolic blood pressure (DBP) from baseline in both hypertensive and pre-hypertensive individuals [11–13] and to improve plasma lipid profiles in both normo-lipidaemic and hypercholesterolaemic subjects [11, 13–15]. OLE has also been found to induce acute reductions in arterial stiffness compared to a control by our research group [16], which agrees with data suggesting that OO significantly improves vascular function [17–19] and blood pressure [20] and these improvements are specifically associated with phenolic-rich rather than phenolic-poor OO [21]. In contrast, however, other studies have demonstrated that OLE supplementation has no effect on plasma lipids [12, 22], ambulatory blood pressure (ABP), cytokines or carotid intima-media thickness [22].

In order to better understand the impact of OLE intake, and to address the inconsistent existing data, the current randomised, controlled, double-blind, crossover intervention trial was designed to examine the effect of OLE on 24-h ambulatory blood pressure (BP) and a range of related vascular, lipid and inflammatory markers in 60 pre-hypertensive male volunteers.

## Methods

### Subjects and screening

A chronic human study was performed at the School of Food and Nutrition, Massey University, Auckland, New Zealand, from May–September 2013. The primary outcome measure was BP. Secondary outcome measures were vascular function, arterial stiffness, plasma lipids, glucose, insulin, fructosamine, oxidised LDL, C-reactive protein (CRP), adiponectin, cell adhesion molecules and cytokines.

### Power calculation

A mean reduction of 5mmHg in SBP was chosen as a clinically significant end point, since at population level this has been estimated to relate to a 20 % reduction in CVD morbidity and mortality [23]. To observe such a reduction using a crossover trial design with a standard deviation of 12.5 mmHg in a normal population would require 50 subjects to be longitudinally studied with 90 % power and a significance value of 0.05. Eleven extra subjects were enrolled to allow for dropouts.

Volunteers were recruited for the study through advertisements placed in local newspapers in the Auckland area and via flyers and posters on the Massey University campus as well as in shops and community buildings in the surrounding area. Email advertisements were sent to Massey University students and staff and those belonging to the IFNHH nutrition unit volunteer database. Individuals who responded to advertisements were asked to complete a health and lifestyle questionnaire online or by telephone. Those who fitted the inclusion criteria were invited to the clinical unit for assessment of further inclusion/exclusion criteria. Suitable subjects were pre-hypertensive, non-smoking males, free from chronic disease, including cardiovascular disease, diabetes, cancer, inflammatory or digestive disorders. Asthmatics and those consuming more than 21 U/week of alcohol were excluded. Subjects were not taking anti-hypertensives, statins or other medication or dietary supplements that may affect BP, lipids or blood clotting, including fish oil. Pre-hypertensive subjects were identified as those presenting with average SBP in the range 121–140 mmHg and/or average DBP in the range 81–90 mmHg at screening. Individuals with BP outside of these ranges were excluded. Subjects with food allergies or intolerances and those on a weight-reducing or restrictive diet (including vegetarian and vegan) were also excluded. Subjects arrived for screening fasted, and height and weight were measured using a stadiometer and Tanita weighing scales to calculate BMI. BP was measured after 5-min rest, seated and with the subject’s left arm resting on a table, using an Omron digital BP monitor (HEM-907). Three readings were taken 60 s apart and averaged. Subjects were not permitted to talk during measurements. Mid-upper arm circumference (MUAC) was verified by tape measure in order to select the correct-sized BP cuff. A total of 61 suitable subjects were identified and accepted onto the trial.

### Randomisation and blinding

Order of treatment allocation was done using the website randomization.com using a random block design. Products were labelled by an external individual using four-digit random number codes in identical bottles made from opaque plastic. The treatment codes were kept offsite and not released until statistical analysis was complete. Therefore, allocation concealment was achieved and both researchers and subjects were blinded to which product was being consumed at which time.

### Study design

**Fig. 1:**
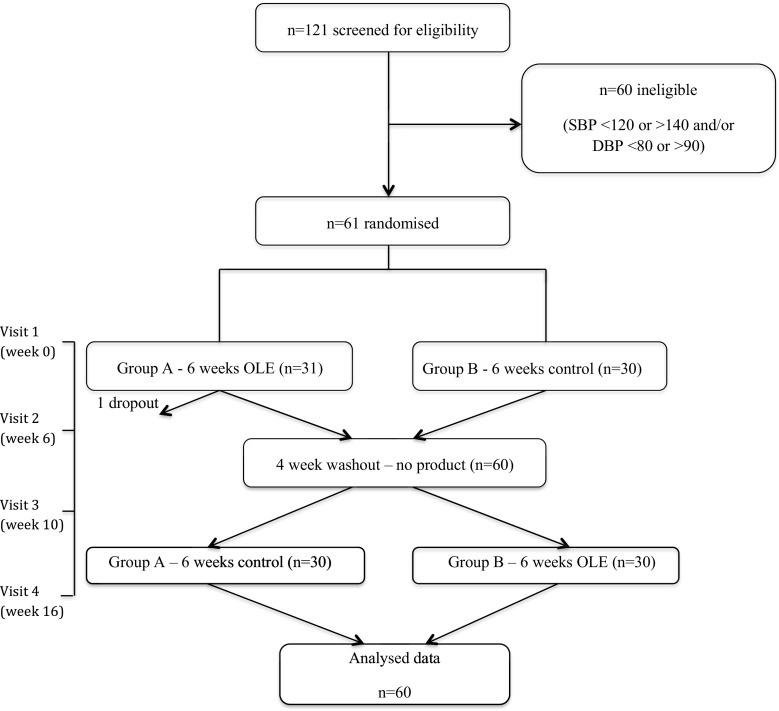
Study flow

The study was a double-blind, randomised, controlled, crossover trial (Australia New Zealand Clinical Trials Registry number: ACTRN12613000180718, Clinicaltrials.gov ID: NCT01796561, see Fig. 1 for study design). This study was conducted according to the guidelines laid down in the Declaration of Helsinki, and all procedures involving human subjects were approved by the University of Reading Research Ethics Committee (UREC 13/02). Written informed consent was obtained from all subjects. Sixty-one subjects aged 24–72 years consumed liquid OLE supplement or a control in a random order twice per day for 6 weeks, separated by a 4-week wash out period, during which no product was consumed. Six weeks was chosen as, in relation to health claims for food products, the European Food Safety Authority states that ‘scientific evidence for the substantiation of health claims on the maintenance of normal blood pressure can be obtained from human intervention studies showing a short-term (e.g. 3–4 weeks) reduction in systolic blood pressure, or a reduction in diastolic blood pressure’ [24]. Subjects avoided plant sterol-/stanol-enriched spreads and all olive-containing products (olives, olive oil, olive margarine, tapenade) for the duration of the study (16 weeks). Clinical visits took place at weeks 0, 6, 10 and 16 (before and after consuming each study product, four visits in total). Subjects refrained from consuming alcohol and taking part in strenuous exercise the day before study visits. The evening before study visits subjects consumed a standard low-fat meal of low phenolic content that was provided to participants (Weight Watchers macaroni cheese).

### Intervention

The study product, ‘Olive leaf extract, extra strength’, is a commercially available, concentrated OLE liquid product manufactured by Comvita, Limited (Paengaroa, Te Puke, New Zealand) and comprises vegetable glycerine and water in a 50:50 ratio plus olive leaf extract. The commercial product is standardised to contain between 6.6 and 7.9 mg oleuropein/ml; the batch used in this study contained 6.81 mg oleuropein/ml and 0.32 mg HT/ml, providing 136.2 mg oleuropein and 6.4 mg HT per day. The full phenolic profile can be found in Table 1.

**Table 1:** Phenolic composition of study product. Analysis performed via UPLC by product manufacturer (Comvita, Limited)

Phenolic compound	mg/ml
Oleuropein	6.81
Oleoside	0.73
Hydroxytyrosol	0.32
Luteolin-7-*O*-glucoside	0.17
Tyrosol	0.12
Verbascoside	0.09
Apigenin-7-*O*-glucoside	0.07
Rutin	0.02
Vanillic acid	0.01
Vanillin	0.01
Luteolin	0.01

Subjects were instructed to consume 10 ml, twice per day, with food (20 ml per day in total) and were supplied with measuring cups and spoons. The control product comprised vegetable glycerine and water in a 50:50 ratio plus commercially available food colourings and flavourings in safety-approved quantities in order to match OLE as closely as possible on appearance, taste, texture and aroma.

### Clinical visits

Subjects arrived for all clinical visits in a 12-h fasted state. Temperature was measured with an inner ear thermometer (Braun Thermoscan 6014) to check for the presence of acute infections. Subjects rested supine for 10 min before vascular function measurements began. Measurements were taken in a quiet room by a single trained operator. The SphygmoCor (AtCor Medical, Sydney) was used to measure pulse wave velocity (PWV). Pulse wave velocity has been validated and shown to be an independent predictor of cardiovascular mortality and morbidity [25]. The velocity at which pulse waves travel along arteries is dependent on the elasticity of the artery walls. PWV measures the amount of time taken for pressure waves to travel over a known distance and is calculated as the distance between the two positions of the pulse transducer divided by the time delay measured between pressure upstroke at each site. Here, PWV was recorded between carotid and femoral artery sites. The pressure wave was recorded directly by means of a high-fidelity applanation tonometer which is a force recorder. The less elastic, or more stiff, the artery walls, the faster the velocity and the higher the PWV value [26]. Pulse wave analysis (PWA) is a non-invasive method which measures augmentation index (AIx), a manifestation of arterial stiffness. The method is sensitive to acute effects such as the ingestion of red wine [27] and black and green tea [28]. AIx is strongly correlated with PWV [29]. PWA was performed using the SphygmoCor, whereby a hand-held tonometry probe was used to flatten the subject’s radial artery. The SphygmoCor takes a 10-s snapshot of the arterial pressure wave and derives the ascending aortic pressure wave, measuring AIx. AIx indicates the augmentation of the incident pulse wave due to the reflection and is found by taking the difference between the first and second derived aortic systolic peaks as a percentage of pulse pressure. AIx has been found to be influenced by heart rate [30] and so an index normalised for a heart rate of 75 bpm (AIx@75) was used here. Body composition was assessed via bioelectrical impedance using an InBody 230 analyser. Blood samples obtained via single venepuncture were collected into heparin and EDTA vacutainers (BD).

### Compliance measures

Subjects were asked to return all remaining bottles of study product at the end of each intervention period. Remaining liquid supplement was weighed and recorded. Subjects were asked to complete weekly online questionnaires and supplied with daily tick sheets.

### Blood pressure

Twenty-four-hour ABP was assessed at weeks 0, 6, 10 and 16 using automated monitors (Model TM-2430, Scan Med, A&D Medical, UK). Devices were programmed to measure BP every 30 min between the hours of 0700 and 2200 and every 60 min between the hours of 2200 and 0700 with the cuff located on the upper left arm. In order to collect accurate data for a 24-h period, subjects were asked to wear the monitors for a duration of 25 h, and the first two readings following fitting of the device were excluded to allow for subjects to adjust to the presence of the machine. Subjects were asked to refrain from strenuous exercise whilst wearing the monitor and to keep the device on for the entire 25-h period, apart from when showering and dressing, which was to be done between measurements. Subjects were instructed to use the BP monitors on a similar type of day to standardise for activity level, in particular to account for differences that may arise due to a work or non-work day. Subjects completed an activity diary and recorded sleep and wake times, which were used to classify data into ‘night’ and ‘day’ periods. Here, data collected within night and day periods were averaged, and an average of the whole 24-h period was also calculated.

### Biochemical measures

Blood collected in EDTA and heparin vacutainers was centrifuged at 1550×*g* for 15 min to separate plasma. Plasma was stored in low-binding Eppendorf tubes (Axygen, Tewksbury MA, USA) at −80 °C until analysis. Total cholesterol (TC), HDL cholesterol (HDL-C), triglycerides (TAG), glucose and insulin were measured at North Shore Hospital, Auckland, in a Dimension Vista 1500 Intelligent Lab System. LDL cholesterol (LDL-C) was calculated using the Friedewald formula: [(LDL-C) = (TC) − (HDL-C) – (TAG)/2.2]. QUICKI was calculated using the formula: 1/(log insulin + log glucose). HOMA-IR was calculated using the formula: (glucose × insulin)/22.5. Fructosamine was measured using a colorimetric assay (Roche Cobas, Indianapolis, USA). Vascular cell adhesion molecule-1 (VCAM-1), intercellular adhesion molecule-1 (ICAM-1), E-selectin, P-selectin, CRP and adiponectin were analysed using multiplex Luminex kits following standard instructions provided by the manufacturer (R&D). IL-6, TNF-α, IL-1-β, IL-10 and IL-8 were measured in a random subset of subjects (mean age 42.3 ± 13.1 years) using an ultrasensitive multiplex Luminex kit (R&D). Oxidised LDL was measured in duplicate via an ELISA kit (Mercodia, Sweden). Plasma samples were analysed for nitrite and nitrate using chemiluminescence. Briefly, samples and standards containing nitrite and nitrate were first reduced to NO, which was then quantified using an NO analyser (NOA Eco Physics chemiluminescence detector, model 88 et). To determine total nitrite and nitrate concentrations, collectively termed ‘NOx’, samples were added to 0.1 mol/L vanadium (III) chloride in 1 M hydrochloric acid refluxing at 90 °C. Nitrite concentrations were determined by addition of samples to 1.1 % potassium iodide in glacial acetic acid under nitrogen at room temperature. Concentrations of nitrate were calculated by subtraction of nitrite from NOx values.

### Statistical analysis

Statistical analysis was performed using SPSS statistics software version 21 (IBM) for data in which a complete set of four values (one per clinical visit) was available for a subject per variable. Data were checked for normality using the Kolmogorov–Smirnov and Shapiro–Wilk tests. Order of treatment effects was assessed using 2-way analysis of variance. Where there were no group × treatment interactions present, whole group data were analysed. Significant group × treatment effects were indicated for fructosamine and IL-6 and so only data from visits 1 and 2 were used for these variables. Data were grouped by treatment, and difference values were calculated by subtracting the baseline values from the end of treatment period values. Difference values from the two treatments were compared to each other using a paired Student’s *t* test. *p* values <0.05 were deemed statistically significant.

## Results

### Compliance

One subject withdrew from the study after the first clinical visit due to relocation. There were no other dropouts, and 60 subjects completed the study. Weighing of the remaining liquid OLE product revealed a compliance rate of 70.19 % (±SD 17.72) to OLE and 74.54 % (±SD 18.28) to the control. One subject experienced acne, and four subjects experienced mild stomach upset during the course of intervention, but these symptoms were also experienced by subjects on the control and did not result in subject withdrawal from the study.

### Baseline characteristics of the subjects

All subjects were free from diagnosed chronic disease with a mean daytime BP of 139/83 mmHg (Table 2), classifying them as prehypertensive [31]. Average BMI was 26.7 kg/m^2^, although subjects ranged from 20.4 to 37.4 kg/m^2^ (healthy to obese). Subjects had total cholesterol, LDL-C and TC/HDL-C levels above that considered physiologically normal (5 mmol/L, 3.1 mmol/L and 4.3 vs. guideline values of <4  mmol/L, <2 mmol/L and <4, respectively [32]) (Table 2). Triglyceride and HDL-C concentrations were within the normal range (1.4 and 1.3 mmol/L vs. guideline values of <1.7 and ≥1 mmol/L).

**Table 2:** Baseline characteristics of the subjects

Variable	Mean (SD)
Age (years)	45.3 (±12.7)
BMI (kg/m^2^)	27.0 (±3.4)
% body fat	22.5 (±6.6)
Total cholesterol (mmol/L)	5.0 (±1.0)
LDL cholesterol (mmol/L)	3.1 (±0.9)
HDL cholesterol (mmol/L)	1.3 (±0.4)
Triglycerides (mmol/L)	1.4 (±0.9)
TC/HDL cholesterol ratio	4.3 (±1.7)
Glucose (mmol/L)	5.2 (±0.4)
Insulin (mU/L)	8.9 (±5.8)
24-h SBP/DBP (mmHg)	135 (±11)/81 (±8)
Daytime SBP/DBP (mmHg)	139 (±12)/83 (±9)
Night time SBP/DBP (mmHg)	116 (±10)/68 (±8)

*BMI* body mass index, *LDL* low-density lipoprotein, *HDL* high-density lipoprotein, *TC* total cholesterol, *SBP* systolic blood pressure, *DBP* diastolic blood pressure

### Blood pressure and vascular function

Twenty-four-hour SBP, 24-h DBP and daytime SBP and DBP were all significantly reduced following intake of the OLE relative to control due to a decrease in BP after OLE consumption and an increase in BP after the control (Table 3). There were no significant differences between the effects of the two treatments on night time BP (Table 3). No significant impact of OLE on PWV was detected, although there was a tendency for OLE to attenuate an increase in PWA-AI@HR75 after consumption of the control (*p* = 0.071) (Table 4).

**Table 3:** Effect of OLE on 24-h ambulatory blood pressure data

Variable (mmHg)	OLE			Control			OLE versus control mean difference (SD)	*n* ^§^	*p* ^‡^
	Baseline	End	Mean change (SD)	Baseline	End	Mean change (SD)			
24-h SBP	134.75 (±11.67)	133.27 (±9.45)	−1.49 (±7.56)	132.59 (±10.28)	134.45 (±10.92)	1.84 (±7.90)	−3.33 (±10.81)	45	0.045
24-h DBP	80.35 (±8.65)	80.03 (±8.09)	−0.20 (±5.65)	79.10 (±8.11)	81.25 (±8.91)	2.22 (±6.32)*	−2.42 (±7.61)	45	0.039
Day SBP	139.69 (±12.28)	137.45 (±10.21)	−2.25 (±8.01)	137.49 (±10.25)	139.22 (±11.30)	1.70 (±7.13)	−3.95 (±11.48)	44	0.027
Day DBP	83.71 (±8.86)	83.29 (±8.12)	−0.50 (±6.13)	82.32 (±8.40)	84.97 (±8.95)	2.50 (±6.50)*	−3.00 (±8.54)	44	0.025
Night SBP	115.58 (±10.22)	115.78 (±9.67)	0.16 (±10.44)	112.89 (±9.98)	116.16 (±11.52)	3.19 (±9.68)*	−3.02 (±14.41)	43	0.176
Night DBP	67.66 (±8.53)	67.43 (±7.52)	−0.09 (6.81)	66.24 (±7.43)	67.81 (±7.95)	1.47 (±6.12)	−1.56 (±9.69)	43	0.298

Data points are mean ± SD

*OLE* olive leaf extract, *SBP* systolic blood pressure, *DBP* diastolic blood pressure

* End value was significantly different from baseline value, paired Student’s *t* tests

^§^Missing data points result predominantly from unreturned measurements by volunteers

^‡^Values derived from paired Student’s *t* tests comparing OLE mean change values with control mean change values

**Table 4:** Effect of OLE on vascular function measures

Variable	OLE			Control			OLE versus control mean difference (SD)	*n*	*p* ^‡^
	Baseline	End	Mean change (SD)	Baseline	End	Mean change (SD)			
PWV (m/s)	9.35 (±1.94)	9.06 (±1.54)	−0.29 (±1.41)	9.45 (±1.71)	8.97 (±1.86)	−0.48 (±1.58)*	0.19 (±1.91)	54	0.461
PWA-AI@HR75 (%)	10.19 (±12.67)	11.37 (±12.28)	1.19 (±5.92)	9.54 (±11.96)	12.94 (±10.62)	3.41 (±3.41)*	−2.22 (±8.86)	54	0.071

Data points are mean ± SD

*OLE* olive leaf extract, *PWV* pulse wave velocity, *PWA-AI@HR75* pulse wave analysis-derived augmentation index corrected to a heart rate of 75 bpm

* End value was significantly different from baseline value, paired Student’s *t* tests

^‡^Values derived from paired Student’s *t* tests comparing OLE mean change values with control mean change values

### Biochemical analysis and body composition

OLE intake significantly reduced plasma TC, LDL-C and TAG from baseline. Overall reductions relative to the control were 0.32, 0.19 and 0.18 mmol/L, respectively (Table 5). HDL-C significantly decreased from baseline following consumption of OLE; however, there were no significant effects on HDL-C or the LDL-C/HDL-C ratio compared to the control. There was a near-significant decrease in total cholesterol/HDL-C ratio following OLE consumption (*p* = 0.055) compared to the control (Table 5). There were no effects of OLE intake on fasting glucose, insulin, fructosamine or calculated HOMA-IR or QUICKI indices (Table 6). Similarly there was no effect on oxidised LDL, CRP, adiponectin, ICAM-1, VCAM-1, P-selectin, E-selectin, IL-6, IL-10, IL-1β or TNF-α (Table 7). However, OLE significantly reduced plasma IL-8 compared to the control (*p* < 0.05) (Table 7). There was no significant difference in plasma nitrite between the two groups (olive leaf change from baseline: 71.6 nM ± 801, *n* = 38; control change from baseline: 114 nM ± 732, *n* = 38). There was no impact of either treatment on body composition (see Supplementary Material Table S1).

**Table 5:** Effect of OLE on fasting plasma lipids

Variable	OLE			Control			OLE versus control mean difference (SD)	*n*	*p* ^‡^
	Baseline	End	Mean change (SD)	Baseline	End	Mean change (SD)			
Total cholesterol (mmol/L)	5.11 (±0.99)	4.78 (±0.99)	−0.33 (±0.47)*	5.02 (±1.02)	5.00 (±0.97)	−0.01 (±0.44)	−0.32 (±0.70)	52	0.002
LDL-C (mmol/L)	3.14 (±0.88)	2.94 (±0.87)	−0.20 (±0.41)*	3.09 (±0.90)	3.08 (±0.89)	−0.01 (±0.37)	−0.19 (±0.56)	52	0.017
HDL-C (mmol/L)	1.29 (±0.40)	1.24 (±0.34)	−0.05 (±0.18)*	1.30 (±0.38)	1.29 (±0.38)	−0.007 (±0.17)	−0.04 (±0.24)	52	0.202
TAG (mmol/L)	1.48 (±0.87)	1.30 (±0.77)	−0.18 (±0.46)*	1.39 (±0.88)	1.40 (±0.84)	0.004 (±0.51)	−0.18 (±0.48)	52	0.008
LDL-C/HDL-C ratio	2.68 (±1.18)	2.58 (±1.12)	−0.09 (±0.42)	2.61 (±1.16)	2.63 (±1.16)	0.02 (±0.39)	−0.12 (±0.54)	52	0.125
TC/HDL-C ratio	4.32 (±1.72)	4.16 (±1.63)	−0.16 (0.54)*	4.22 (±1.73)	4.24 (±1.74)	0.03 (±0.44)	−0.19 (±0.70)	52	0.055

Data points are mean ± SD

*OLE* olive leaf extract, *LDL-C* low-density lipoprotein cholesterol, *HDL-C* high-density lipoprotein cholesterol, *TAG* triglycerides, *TC* total cholesterol

* End value was significantly different from baseline value, paired Student’s *t* tests

^‡^Values derived from paired student’s *t* tests comparing OLE mean change values with control mean change values

**Table 6:** Effect of OLE on measures of glucose metabolism

Variable	OLE			Control			OLE versus control mean difference (SD)	*n*	*p* ^‡^
	Baseline	End	Mean change (SD)	Baseline	End	Mean change (SD)			
Glucose (mmol/L)	5.32 (±0.44)	5.27 (±0.54)	−0.05 (±0.37)	5.28 (±0.46)	5.33 (±0.43)	0.05 (±0.32)	−0.10 (±0.52)	52	0.163
Insulin (mU/L)	9.46 (±6.1)	8.86 ±(5.25)	−0.60 (±4.50)	9.27 (±5.73)	9.97 (±7.49)	0.70 (±5.15)	−1.30 (±7.19)	52	0.197
HOMA-IR	2.28 (±1.57)	2.13 (±1.45)	−0.15 (±1.18)	2.25 (1.64)	2.42 (1.96)	0.17 (±1.21)	−0.32 (±1.80)	52	0.483
QUICKI	0.63 (±0.10)	0.64 (±0.09)	0.01 (±0.07)	0.63 (±0.11)	0.63 (±0.12)	−0.002 (±0.07)	0.01 (±0.10)	52	0.482
Fructosamine^§^ (µmol/L)	230.41 (±18.04)	221.24 (±15.94)	−9.17 (±13.19)	229.29 (±14.30)	222.48 (±15.17)	−6.81 (±14.82)	−2.36	29, 31	0.517

*OLE* olive leaf extract, *HOMA-IR* homoeostasis model assessment-estimated insulin resistance, *QUICKI* quantitative insulin sensitivity check index

^‡^Values derived from paired Student’s *t* tests comparing OLE mean change values with control mean change values

^§^Significant treatment × sequence interactions were shown for this variable; therefore, only data derived from the first intervention period (week 0–week 6) are reported

**Table 7:** Effect of OLE on markers of inflammation

Variable	OLE			Control			OLE versus control mean difference (SD)	*n*	*p* ^‡^
	Baseline	End	Mean change (SD)	Baseline	End	Mean change (SD)			
oxLDL (U/L)	72 (±24)	69 (±22)	−3.2 (±17)	69 (±25)	72 (±31)	2.7 (±18)	−5.8 (±26)	50	0.124
CRP (ug/ml)	1.0 (±0.63)	1.0 (±0.80)	0.01 (±0.82)	1.2 (±1.4)	0.9 (±0.85)	−0.3 (±1.3)	0.3 (±1.6)	50	0.585
Adiponectin (ug/ml)	6 (±1.7)	6.3 (±1.7)	0.1 (±1.3)	6.5 (±1.8)	6.4 (±1.8)	−0.2 (1.1)	0.3 (±1.6)	50	0.218
ICAM (ng/ml)	102 (±36)	104 (±40)	2.4 (±16.3)	105 (±41)	105 (±39)	0.2 (±14)	2.1 (±21)	51	0.474
VCAM (ng/ml)	701 (±196)	727 (±223)	26 (±121)	723 (±204)	721 (±198)	−1.3 (±131)	27 (±212)	50	0.372
P-selectin (ng/ml)	40 (±11)	41 (±9.7)	0.7 (±6.8)	41 (±11)	41 (±10)	0.6 (±5.3)	0.1 (±7.7)	50	0.924
E-selectin (ng/ml)	42 (±14)	43 (±13)	0.6 (±9.0)	42 (±12)	43 (±14)	1.0 ± (6.7)	−0.4 (±12.7)	52	0.842
IL-1β (pg/ml)	2.5 (±0.75)	2.4 (±0.75)	−1.0 (±0.38)	2.5 (±0.83)	2.5 (±0.78)	0.0 (±0.34)	−0.1 (±0.53)	20	0.270
IL-6^§^ (pg/ml)	2.1 (±1.0)	2.3 (±0.92)	0.3 (±0.52)	2.0 (±0.94)	2.1 (±1.2)	0.1 (±0.69)	0.1	8, 11	0.671
IL-8 (pg/ml)	2.7 (±1.3)	2.1 (±1.2)	−0.7 (±0.84)*	2.5 (±1.4)	2.4 (±1.1)	−0.0 (±0.73)	−0.6 (±1.1)	19	0.026
TNF-α (pg/ml)	6.5 (±2.0)	6.2 (±2.0)	−0.3 (±0.70)	6.1 (±2.1)	6.3 (±1.9)	0.2 (±0.72)	−0.5 (±1.3)	18	0.101
IL-10 (pg/ml)	1.1 (±0.57)	1.2 (±0.59)	0.1 (±0.30)	1.2 (±0.59)	1.2 (±0.75)	0.0 (±0.36)	0.1 (±0.46)	19	0.594

Data points are mean ± SD

*OLE* olive leaf extract, *oxLDL* oxidised LDL, *CRP* C-reactive protein, *ICAM* intercellular adhesion molecule, *VCAM* vascular cell adhesion molecule, *IL* interleukin, *TNF* tumour necrosis factor

* End value was significantly different from baseline value, paired Student’s *t* tests

^‡^Values derived from paired Student’s *t* tests comparing OLE mean change values with control mean change values

^§^Significant treatment × sequence interactions were shown for this variable; therefore, only data derived from the first intervention period (week 0–week 6) are reported

## Discussion

Previous studies have indicated potential blood pressure and lipid-lowering effects of OLE in humans, but results have thus far lacked consistency, perhaps due to differences in phenolic dose, duration and study design. Here, we provide data demonstrating that OLE has the potential to significantly reduce 24-h and daytime SBP and 24-h and daytime DBP relative to control. The magnitude of BP changes observed here (SBP by 3.33 and 3.95 mmHg and DBP by 2.42 and 3.00 mmHg (24 h and daytime values, respectively)) can be considered physiologically significant. Data from observational studies suggest that 2 mm Hg reductions in SBP and DBP are associated with 6% and 7 % reductions in CHD risk and 10% and 15 % reductions in stroke and heart attack respectively [33, 34]. Extrapolating from this would suggest that regular OLE intake may be associated with a 9–14 % reduction in CHD risk and a 20–22.5 % reduction in risk of stroke and heart attack.

It has been postulated that oleuropein is the key hypotensive component of OLE due to L-type Ca^2+^ channel antagonistic effects [35, 36]. In addition, verbascoside has been demonstrated to inhibit angiotensin-converting enzyme in vitro [37] as has oleacein [38]. With respect to oleuropein, our intervention provided 136 mg/day, compared to 200 mg/day used in two previous studies; which resulted in mean reductions in systolic and diastolic blood pressure of 13 and 5 mmHg, respectively, in pre-hypertensive MZ twins [11] and 12 and 5 mmHg, respectively, in hypertensive patients [13] a magnitude of effect similar to that of Captopril, a common anti-hypertensive drug [13]. In a further study, a dose of 51 mg oleuropein/day induced no significant reductions in BP [22] although this study tested OLE capsules, which may be less bioavailable than the liquid used in the current study [39]. Assuming linearity between dose and BP reductions, our prescribed dose would be expected to yield a reduction of approximately 8 mmHg, higher than that measured in our study. However, as our compliance rate was 70.19 % with respect to OLE consumption, daily oleuropein intake can be estimated to be lower at around 95 mg oleuropein per day. Furthermore, we employed 24-h ambulatory BP measures in our study, which arguably provide more robust information on BP compared to the single measures used in the aforementioned studies [40]. Studies examining the effects of OO phenolics and their metabolites suggest that these may influence NO production in vivo [41], or scavenge ROS in the vasculature [42], following their appearance in the circulation. Whilst previous studies have linked the phenolic content of OO with increases in nitric oxide and ultimately clinical outcomes [20, 43], we observed no significant impact on circulating nitrites. It is possible that nitrite contamination from the use of samples collected in EDTA tubes lead to the high standard deviation of this data and masked any changes that occurred. Additionally, it is noteworthy that the product used here has not been completely characterised meaning that other bioactives aside from polyphenols, such as minerals, squalene and, triterpenoids such as oleanolic, ursolic and maslinic acids [44], could have been responsible for the observed blood pressure effects, thus pointing towards a different mechanism of action besides NO. For example, African olive leaf cultivars which are triterpenoid-rich and polyphenol-poor have been reported to prevent hypertension and atherosclerosis and improve insulin resistance in Dahl salt-sensitive rats [45].

With arguably more pronounced effects than on BP, OLE intake was also associated with physiologically significant reductions in TC, LDL-C and TAG of 0.32, 0.19 and 0.18 mmol/L, respectively, when compared to the control, with no detrimental effect on HDL-C. Considering previous trials conducted with statins, the TC and LDL-C reductions reported in the present study could equate to an overall CVD risk reduction of 4.2 % [46] and 9.75 % [47], respectively. Similarly, data from a meta-analysis of population-based prospective cohort studies report that a 1 mmol/L increase in TAG results in a 32 % CHD risk increase. On this basis, consumption of OLE at the dose provided in our study may promote a 5.76 % CHD risk reduction [48]. Data regarding the effects of OLE on plasma lipids have been somewhat inconsistent. For example, in a study of 20 MZ twin pairs, a 200 mg/day intake of oleuropein resulted in a 0.6 mmol/L decrease in TC, a 0.4 mmol/L decrease in LDL cholesterol and no change in TAG relative to healthy lifestyle advice alone after 8 weeks, whilst a 100 mg/day dose had no significant effects on lipids [11], whereas a larger study (*n* = 148) found less efficacious changes of −0.15 mmol/L in TC, −0.10 mmol/L in LDL-C and −0.13 mmol/L in TAG [13]. A more recent study reported decreases of 0.68, 0.90 and 0.047 mmol/L in TC, LDL-C and TAG, respectively (with a non-significant increase in HDL-C), after 12 months consumption of a supplement containing 100 mg oleuropein [15], providing some evidence of sustained and larger effects over longer periods of time. Individual differences in the absorption and metabolism of OLE phenolics could be responsible [39].

The mechanisms underlying the lipid-lowering effects of OLE are presently unknown. However, animal data suggest that the consumption of phenolic components of OLE appears to decrease the activities of key cholesterol-regulatory enzymes, 3-hydroxy- 3-methylglutaryl-CoA (HMG-CoA) reductase (the main target of statins) and acetyl-CoA cholesterol acyltransferase (ACAT), resulting in decreased cholesterol biosynthesis [49]. Additional animal data suggest that olive phenolics may impact on bile flow, increasing biliary cholesterol and bile acid concentrations, leading to their increased faecal excretion [50]. Interestingly, a recent paper reporting favourable modification of lipid profiles by OLE [15] also observed osteoblast stimulation and hypothesised that as osteoblasts and adipocytes derive from the same mesenchymal stem cells, this may explain the change in lipid profiles. Once again, there is evidence to suggest that non-phenolic components may contribute to lipid-lowering effects [51].

Chronic OLE intake reduced plasma IL-8 concentration in a subgroup of the subjects (*n* = 19). Due to the high natural variability of cytokine production, greater power may be required for reliable and meaningful data [52]. When accounting for multiple comparisons of inflammatory markers, the result is no longer significant (a *p* value < 0.004 would be needed to be statistically significant [0.05/12 comparisons (12 inflammatory markers)]; however, the finding reflects our previous data, indicating that an acute dose of OLE decreases ex vivo production of LPS-stimulated IL-8 (but not other cytokines) in whole blood cultures [16]. Anti-inflammatory effects of OLE are also indicated by its use in a patented haemorrhoid treatment [53], and from data demonstrating that OLE phenolics reduce inflammatory cytokines in animal [54] and ex vivo [55] studies [22]. IL-8 is associated with increased risk of future CVD incidences [56], perhaps through its ability to destabilise existing atherosclerotic plaques by down-regulation of tissue inhibitors of metalloproteinase expression [57]. However, cytokines lack the robustness of other CVD biomarkers such as blood pressure and plasma lipids, and it is difficult to attribute clinical importance to reductions in these markers [58].

The effect of OLE on glycaemic control was worthy of investigation as chronic OLE supplementation has been related to improvements in an oral glucose tolerance test and additionally 1 g olive leaf fed with 300 g white rice has been observed to significantly reduce blood glucose at 30 and 60 min in borderline diabetics [22, 59]. In both instances, these effects were thought to be mediated by the inhibitory action of OLE polyphenols on intestinal and/or salivary α-amylases; however, it is also possible that OLE aglycones compete with glucose released from food in the gut for glucose receptors, resulting in less absorption. In the present study, there were no significant effects of OLE on fasting glucose, insulin, fructosamine, QUICKI or HOMA-IR indices. In line with this, a previous study has indicated no change in fasting glucose after 3-week VOO supplementation compared to refined OO [60].

## Conclusion

The present study has strengthened the existing body of evidence that OLE has the potential to favourably modify blood pressure and plasma lipid profiles. The magnitude of the risk-lowering potential we describe could have significant impact at population level in countries with high prevalence of CVD. The impact of dietary factors towards CVD risk has informed the provision of a diet rich in fruit and vegetables in the primary prevention of hypertension and raised cholesterol [61, 62]. In the near future, there may be enough evidence for this advice to be extended to include phenolic-rich foods. Daily consumption of OLE can result in favourable improvements in several CVD risk factors which could result in a moderate but nonetheless significant reduction in risk, making it a useful addition to a healthy diet and lifestyle.

## Electronic supplementary material

Below is the link to the electronic supplementary material.

Supplementary material 1 (DOCX 18 kb)
