# Supplementary material for: Impact of phenolic-rich olive leaf extract on blood pressure, plasma lipids and inflammatory markers: a randomised controlled trial
Source: Eur J Nutr. 2016 Mar 7;56(4):1421–32. doi: 10.1007/s00394-016-1188-y (PMC5486627; doi:10.1007/s00394-016-1188-y)
Supplement: Supplementary file 1 — Supplementary material 1 (DOCX 18 kb) [file 394_2016_1188_MOESM1_ESM.docx]

# Article title: Impact of phenolic-rich olive leaf extract on blood pressure, plasma lipids an inflammatory markers – a randomised controlled trial

# Journal name: European Journal of Nutrition

Author names: Authors: Stacey Lockyer^1^, Ian Rowland^1^, Jeremy Paul Edward Spencer^1^, Parveen Yaqoob^1^, Welma Stonehouse^2, 3^

Affiliations:

^1^Hugh Sinclair Unit of Human Nutrition, Department of Food and Nutritional Sciences, University of Reading, Berkshire, RG6 6AP, UK

^2^School of Food and Nutrition, College of Health, Massey University, New Zealand

^3^Food and Nutrition Flagship, Commonwealth Scientific Industrial Research Organisation (CSIRO), Adelaide, Australia.

Corresponding author: Professor Jeremy Paul Edward Spencer; address: Hugh Sinclair Unit of Human Nutrition, Department of Food and Nutritional Sciences, University of Reading, Berkshire, RG6 6AP, UK; telephone: [+44 (0) 118 378 8724](tel:+44%20(0)%20118%20378%208724), fax: 0118 931 0080

**Table S1: Body composition data derived via bioelectrical impedance**

| Variable | OLE | | | Control | | | OLE vs. Control  Mean difference (SD) | n | p ‡ |
| --- | --- | --- | --- | --- | --- | --- | --- | --- | --- |
|  | Baseline | End | Mean change (SD) | Baseline | End | Mean change (SD) |  |  |  |
| Weight (kg) | 85.14 (± 12.29) | 85.11 (±12.58) | -0.03 (±1.31) | 84.56 (±12.41) | 84.92 (±12.57) | 0.37 (±1.37) | 0.40 (±1.78) | 48 | 0.131 |
| Body fat (kg) | 19.41 (±7.19) | 19.24 (±7.66) | -0.17 (±1.41) | 18.87 (±7.51) | 19.00 (±7.80) | 0.13 (±1.44) | -0.30 (±1.76) | 48 | 0.245 |
| Fat free mass (kg) | 65.73 (±8.52) | 65.87 (±8.53) | 0.14 (±1.37) | 65.68 (±8.36) | 65.92 (±8.47) | 0.24 (±1.60) | -0.10 (±2.10) | 48 | 0.753 |
| BMI (kg/m²) | 26.66 (±3.21) | 26.65 (±3.27) | -0.01 (±0.40) | 26.50 (±3.21) | 26.57 (±3.33) | 0.08 (±0.41) | -0.09 (±0.56) | 48 | 0.277 |
| % body fat | 22.41 (±6.09) | 22.17 (±6.39) | -0.24 (±1.40) | 21.87 (±6.44) | 21.90 (±6.61) | -0.02 (±1.70) | -0.26 (±2.10) | 48 | 0.395 |

‡Values derived from paired student’s t –tests comparing OLE mean change values with control mean change values

BMI, body mass index
